# Testing physiologic monitor alarm customization software to reduce alarm rates and improve nurses’ experience of alarms in a medical intensive care unit

**DOI:** 10.1371/journal.pone.0205901

**Published:** 2018-10-18

**Authors:** Halley Ruppel, Laura De Vaux, Dawn Cooper, Steffen Kunz, Bernd Duller, Marjorie Funk

**Affiliations:** 1 Yale School of Nursing, Yale West Campus, West Haven, Connecticut, United States of America; 2 Yale New Haven Hospital, New Haven, Connecticut, United States of America; 3 Philips Medizin Systeme Böblingen GmbH, Böblingen, Germany; 4 Perpet Production, Stuttgart, Germany; University of Palermo, ITALY

## Abstract

**Background:**

Clinicians in intensive care units experience alarm fatigue related to frequent false and non-actionable alarms produced by physiologic monitors. To reduce non-actionable alarms, alarm settings may need to be customized for individual patients; however, nurses may not customize alarms because of competing demands and alarm fatigue.

**Objective:**

To examine the effectiveness and acceptance of physiologic monitor software to support customization of alarms.

**Methods:**

This pre/post intervention study was conducted in a 56-bed medical intensive care unit. IntelliVue^®^ Alarm Advisor customization support software for alarm limit violations was installed on all monitors and education on its use provided. For 2 months before and after implementation of the software, data were collected on patient characteristics from the electronic health record, alarm counts and duration from the monitoring system, and nurses’ experience of alarms from a survey.

**Results:**

Medium-priority heart rate, respiratory rate, and arterial pressure alarms were significantly reduced after software implementation (9.3%, 11.8%, and 15.9% reduction respectively; p<0.001 for all). The duration of these alarms was also significantly shorter (7.8%, 13.3%, and 9.3% reduction respectively; p<0.05 for all). The number and duration of SpO_2_ alarms did not decrease (p>0.05 for both). Patients post-intervention had worse Glasgow Coma Scale scores (p = 0.014), but otherwise were comparable to those pre-intervention. Nurses reported less time spent on non-actionable alarms post-intervention than pre-intervention (p = 0.026). Also lower post-intervention were the proportions of nurses who reported that alarms disturbed their workflow (p = 0.027) and who encountered a situation where an important alarm was ignored (p = 0.043). The majority (>50%) agreed that the software supported setting appropriate alarm limits and was easy to use.

**Conclusion:**

Alarm customization software was associated with a reduction in alarms. Use of software to support nurses’ recognition of trends in patients’ alarms and facilitate changes to alarm settings may add value to alarm reduction initiatives.

## Introduction

Clinicians in intensive care units (ICUs) experience alarm fatigue because of the high numbers of false and non-actionable (true but clinically irrelevant) alarms produced by medical devices, especially physiologic monitors. When fatigued by alarms, clinicians may ignore, silence, or deactivate alarms, which can contribute to missing serious and important changes in a patient’s condition [1, 2]. Some common interventions intended to reduce excessive alarms and alarm fatigue include frequent changes to electrocardiography electrodes and adjusting default monitor configurations to have wider alarm limits and longer alarm delays [3]. For example, some published interventions have changed default premature ventricular contraction (PVC) alarm configurations to off or inaudible, reduced default SpO_2_ low alarm limits to 88%, and increased the delay on SpO_2_ alarms [4–9]. Creating alarm profiles for specific patient populations, like pediatric patients [10], can also help to improve relevance of alarms and eliminate unnecessary alarms.

A multi-faceted approach to managing alarm fatigue is needed because interventions target different types of problematic alarms and are implemented by different members of the healthcare team. Problematic types of alarms include false (invalid) alarms and true alarms that are clinically irrelevant (non-actionable) [11, 12]. Frequent electrode changes target false alarms, whereas widening default alarm configurations may reduce non-actionable alarms. Direct caregivers, like nurses, can be responsible for electrode changes, whereas default alarm configurations can be set by clinical administrators and engineers for an entire unit or institution.

To successfully reduce alarms, interventions must also account for the unique characteristics of ICU patients, workflow, and care environment. ICU patients’ vital signs are variable, not only across patients, but also for individual patients whose conditions can change rapidly in the ICU. To account for these changes, alarm settings may need to be customized frequently for individual patients. For example, when patients return from the operating room they may be tachycardic or hypotensive, warranting different alarm settings than when they stabilize hours or days later. Alarm fatigue interventions that included customization of individual patients’ alarm settings have resulted in alarm reduction [8, 9, 13]. Although research on alarm customization is limited, recent evidence suggests that nurses do not customize heart rate alarms enough to reduce alarm rates [14].

The challenge with customizing alarm settings for specific patients is that customization requires nurses’ ongoing assessment of and attention to alarm settings to ensure their continued relevance. When nurses are affected by alarm fatigue, desensitization to alarms may prevent them from proactively managing alarm settings and recognizing alarm patterns. Aspects of nurses’ workflow in ICUs, such as competing cognitive demands and frequent interruptions, also may make customization of alarm settings difficult. As a result, maintaining situational awareness about alarms can be challenging, and device manufacturers have been called on to help design technology that better supports alarm management [7].

One example of improvements in physiologic monitors to reduce excessive alarms is the use of smart alarm algorithms to draw attention to specific clinical conditions, rather than relying on nurses to recognize patterns in multiple alarms for different vital signs [15]. However, interactive software that supports nurses’ decision-making about alarm management is also needed. Clinical decision support software is an increasingly common component of computerized provider order entry and electronic health record systems, assisting clinicians with synthesizing large amounts of data, recognizing patterns in individual patient data, and determining an evidence-based course of action. Features of clinical decision support software, like alerts based on individual patient data, may be useful for assisting nurses at the bedside customize alarms by helping them appreciate alarm trends over time and suggesting changes to minimize alarms.

An example of this type of software is IntelliVue Alarm Advisor (Philips Medizin Systeme Böblingen GmbH, Böblingen, Germany). Alarm Advisor is a FDA-approved software designed to help nurses maintain awareness about the types and frequencies of alarms that their patients are triggering. The software generates visual notifications on the monitor when repeated alarms for the same violation are triggered and silenced within a specified time period. It does not automatically change or modify any alarm settings on its own, but rather provides the nurse the opportunity to do so.

To our knowledge, support software for alarm customization has not been systematically evaluated for its ability to reduce alarms. Although such software may help increase nurses’ awareness of their patient, we were also concerned that this type of software may intensify “alert fatigue” due to the generation of visual notifications on the monitor. “Alert fatigue” and overriding of alerts are common concerns about clinical decision support software for prescribing of medications [16, 17]. Nurses’ experiences using software that produces alerts to prompt changes to alarm settings must be evaluated. Therefore, the purpose of this study was to examine the effectiveness and acceptance of software to support the customization of physiologic monitor alarms. The specific aims were to examine (1) effectiveness of Alarm Advisor at reducing alarms, (2) changes in nurses’ experience of alarms after using Alarm Advisor, and (3) nurses’ acceptance of Alarm Advisor.

## Methods

### Design, setting, and samples

We used a prospective, pre/post-intervention design, and conducted the study in a medical ICU in an academic medical center. The unit has 130 nurses on staff and 56 beds (15 of which are considered “step-down” beds). The Philips Healthcare PIICix monitoring system is in use for all beds. Multiple alarm reduction strategies have been implemented on this unit over the past several years starting in 2013 with the announcement of the National Patient Safety Goal on clinical alarm safety [18]. In July of 2014, audible alarms from PVCs were defaulted to “off” (they still generate an inaudible visual alert) [4]. In February of 2016, the low limit for oxygen saturation (SpO_2_) was changed from 90% to 88% and the delay for the audible alarm was extended from 10 seconds to 15 seconds. The default settings for alarms in the medical ICU are shown in Table 1. No changes to these default settings were made during the course of this study.

**Table 1:** Default alarm settings in the medical intensive care unit.

Alarm	Default Setting
High heart rate limit	120
Low heart rate limit	50
High respiratory rate limit	30
Low respiratory rate limit	8
Low SpO_2_ limit	88
Arterial blood pressure systolic high	180
Arterial blood pressure systolic low	90
Arterial blood pressure diastolic high	90
Arterial blood pressure diastolic low	50
Mean arterial blood pressure high	110
Mean arterial blood pressure low	70
Premature ventricular contractions / minute	>10

We had 2 samples: patients and nurses. The Alarm Advisor software intervention was implemented on all patient monitors in the unit, so individual patient consent was not obtained. Alarm Advisor is intended to enhance clinicians’ interface with the monitors and did not alter standard monitoring and care of patients. De-identified patient characteristic data were obtained from all patients admitted to the unit during the pre- and post-implementation phases; patients who had opted out of participation in research on admission were excluded.

We used a convenience sample of nurses for participation in the pre- and post-implementation surveys. All nurses working on the unit at the time of data collection were eligible to participate; we did not exclude nurses from survey participation for any reason. The Yale University Institutional Review Board Human Investigation Committee approved the study (IRB Protocol ID 1610018535).

### Intervention

Alarm Advisor generates alerts based on medium priority (“yellow”, “warning”) limit violation alarms for high and low heart rate, SpO_2_, pressures, respiratory rate, and number of PVCs per minute. Alarm Advisor does not include high priority (“red”, “crisis”) alarms (e.g., extreme tachycardia) or alarms that are not limit violations (e.g., irregular heart rate). Alarm Advisor alerts can be generated by 2 types of situations: (1) the number of times the same alarm is silenced within a certain time window (e.g., 5 silences in 60 minutes), or (2) the percentage of a certain amount of time that the alarm limit was violated (e.g., heart rate high limit is violated for >50% of a 60-minute period). The number of silences, percentage of time in alarm, and the time windows are set based on the unit’s needs. We determined the appropriate trigger conditions of Alarm Advisor for this unit based on the analysis of baseline alarm data. Alarm Advisor was set to be triggered by a “silence count” of 5 or >20% of time in the alarm condition within a 30-minute time window.

**Fig 1:**
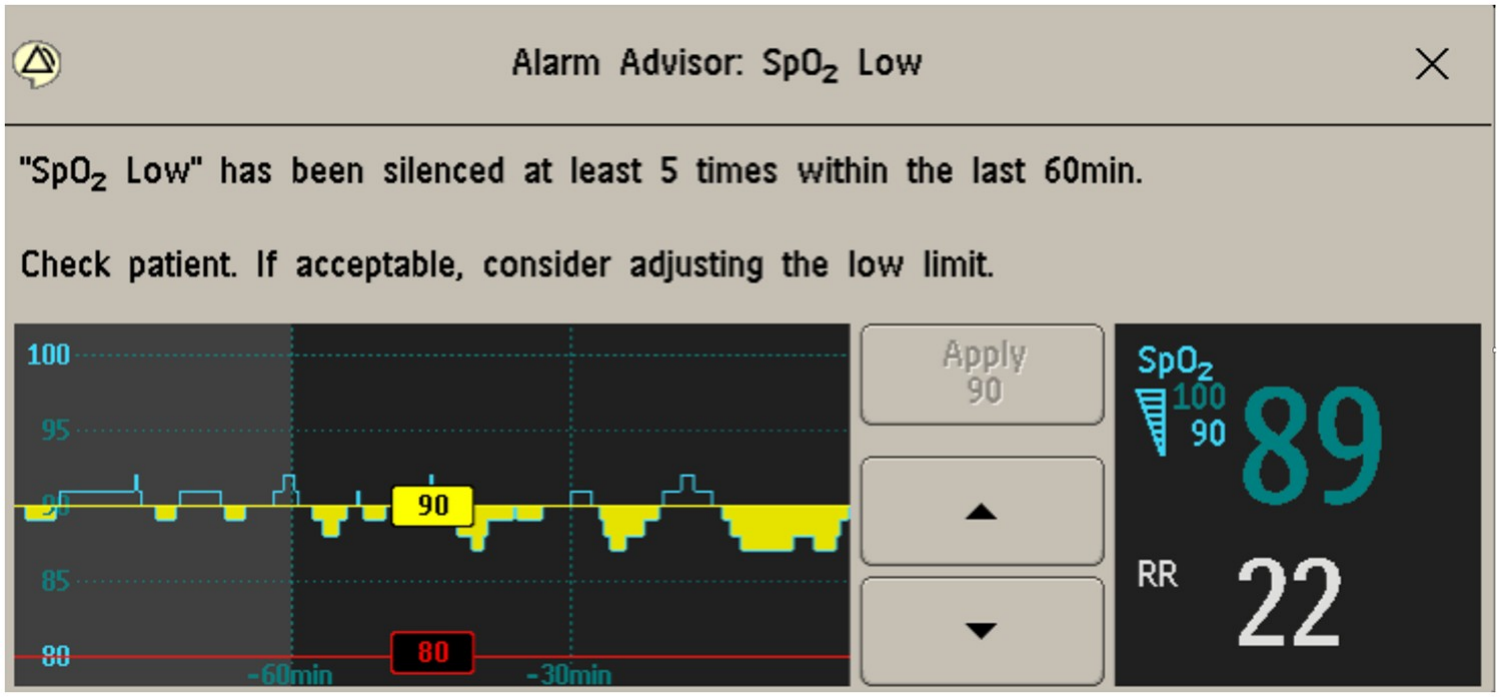
Example Alarm Advisor visual alert for repeated silencing of “SpO_2_ Low” alarms.

Alarm Advisor alerts appear on the bedside monitor screen when someone presses the “silence” button on the patients’ bedside monitor and a trigger condition is fulfilled. Alarm Advisor alerts do not appear if the monitor is silenced outside the room, but a silence outside the room counts toward the violation count. The nurse can adjust the alarm parameter limits from the Alarm Advisor alert window but does not need to take action to be able to close the window. Fig 1 shows an example Alarm Advisor visual alert for a “Low SpO2” alarm that was silenced 5 times in the preceding 60 minutes.

### Data collection

**Fig 2:**
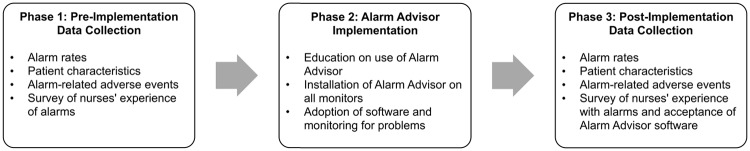
Study design.

The study took place in 3 phases (Fig 2). The first phase occurred before implementation of Alarm Advisor software on the unit. Over 2 months, we collected data to measure alarm rates by exporting raw alarm data from the PIICix system. To evaluate the equivalency of patients before and after the intervention, we collected data on patient characteristics, including age, gender, Glasgow Coma Scale (GCS) scores (as the most appropriate available indicator of the severity of the patient’s condition), and primary diagnosis for all patients admitted to the unit during these 2 months. These data were aggregated from the electronic health record and de-identified for the study by the hospital’s data analytics team. Data of patients who had opted out of research upon admission to the hospital were excluded from the datasets provided to us by the data analytics team. We also monitored for serious adverse events related to alarms by reviewing deaths and resuscitations during the study period for any links to alarm management. We gathered data on nurses’ experiences with alarms using a survey that we developed through review of the literature and expert opinion. We administered the survey to nurses on the unit by distributing cards with the link to the online survey (Qualtrics, Provo, UT). Participation in the survey was voluntary and a statement at the beginning of the survey informed nurses that by taking the survey, they were providing consent to participate in the study. All responses were de-identified.

In the second phase, representatives from Philips Healthcare educated nursing staff in small groups on the use of Alarm Advisor. In an empty patient room on the unit, they discussed the purpose of Alarm Advisor, showed how it worked, and provided opportunities for nurses to use it. In conjunction with hospital-based clinical engineers, they installed Alarm Advisor software on all monitors in the unit. We allowed 2 months for identification of any problems or adverse events and adoption of the software by staff before collecting post-implementation data. We did not identify any problems or adverse events.

During the third phase of the study, we again collected data on alarm rates and patient characteristics and re-administered the survey to nurses. In addition to the questions from the pre-implementation survey, we added 6 questions on user acceptance of Alarm Advisor software. Consistent with phase 1, we collected these data for a 2-month period.

### Data analysis

We performed different analyses for the 3 types of data: patient characteristics, alarms, and nurse surveys. We considered p-values of <0.05 to be statistically significant. To compare patient characteristics before and after implementation of Alarm Advisor, we used the Wilcoxon-Mann-Whitney test for age and GCS and the chi-square test for gender and primary diagnosis.

To analyze the frequency distribution of alarm counts and alarm durations, we used Anderson-Darling tests and quantile-quantile (QQ) plots. Because no particular distribution described these data, we performed a bootstrapping analysis of the means with 10,000 iterations. We present bootstrap means and standard deviations of the means, confidence intervals of the means, and p-values to compare pre- and post-intervention alarm data.

To analyze survey data, we performed chi-square tests to compare the percentage of responses in the pre- and post-implementation phases. For years of medical ICU experience and time spent managing unnecessary alarms, we used the Wilcoxon-Mann-Whitney test.

## Results

### Patient characteristics

Patients in the pre- (N = 677) and post- (N = 659) implementation phases of the study did not differ significantly in age, gender, or the 2 most common primary diagnoses (ICD-10 codes ‘A’ and ‘J’) (Table 2). However, the mean GCS score was significantly lower (worse) in the post-implementation phase (median 14, interquartile range [IQR] 11–15, mean 12.31) than in the pre-implementation phase (median 14, IQR 12–15, mean 12.80) (p = 0.014). There were no alarm-related adverse events in either the pre- or post-implementation phase.

**Table 2:** Patient characteristics pre- and post-intervention.

Characteristic	Pre (N = 677)	Post (N = 659)	p
**Female gender**			0.610^a^
N (%)	308 (45.49%)	309 (46.89%)	
**Age in years**^c^			0.160^b^
Median (IQR)	65 (53–75)	66 (54–77)	
**Glasgow Coma Scale**^d^	N = 533	N = 552	0.014^b^
Median (IQR)	14 (12–15)	14 (11–15)	
Mean	12.80	12.31	
**Primary diagnosis infectious (ICD-10 code ‘A’)**			0.247^a^
N (%)	188 (27.77%)	202 (30.65%)	
**Primary diagnosis respiratory (ICD-10 code ‘J’)**			0.129^a^
N (%)	109 (16.10%)	127 (19.27%)	

IQR, interquartile range; ICD-10, International Classification of Diseases 10^th^ Revision

^a^Chi-square test

^b^Wilcoxon-Mann-Whitney test

^c^Per HIPAA regulations, age 90 or older was aggregated into a single category, so we were unable to obtain mean age

^d^Lower score indicates lower level of consciousness

### Number and duration of alarms

In Table 3, we compare the mean numbers of alarms per bed hour pre- and post-implementation of Alarm Advisor. We present the raw number of alarms in the pre- and post-implementation phases of the study (each of which lasted 2 months), as well as the number of monitoring hours in each phase. The number of monitoring hours was greater in the post-implementation phase than the pre-implementation phase. We then weighted the alarm counts based on the monitoring hours for each parameter, so that we could compare the alarm counts in the 2 phases. Heart rate, respiratory rate, and SpO_2_ were monitored for the same number of hours within each phase because this type of monitoring is standard of care for all patients in the unit, but arterial pressure monitoring occurred for far fewer hours because not all patients had an arterial line. There were many more hours of arterial pressure monitoring in the post-implementation phase (3021 hours) than in the pre-implementation phase (1822 hours), which may be attributed to an increased number and duration of arterial lines during the post-implementation phase.

**Table 3:** Comparison of alarm counts pre-and post-intervention.

	Number of Alarms		Monitoring Hours (2 months per phase)		Weighted Alarm Counts^a^		Bootstrap Means ± SD per Bed Hour		Bootstrap 95% CI of Means		Percent Alarm Reduction	Bootstrap p-value
*Alarm Types / Phase*	*Pre*	*Post*	*Pre*	*Post*	*Pre*	*Post*	*Pre*	*Post*	*Pre*	*Post*		
**All Medium and High Priority**	236507	240242	36545	38205	236507	229804	6.47 ± 0.05	6.29 ± 0.04	6.38–6.56	6.20–6.38	2.8%	0.004
**All High Priority**	38612	39888	36545	38205	38612	38155	1.06 ± 0.02	1.04 ± 0.02	1.02–1.10	1.01–1.08	1.2%	0.666
**All Medium Priority (MP)**	197895	200354	36545	38205	197895	191649	5.41 ± 0.03	5.24 ± 0.03	5.34–5.48	5.18–5.31	3.2%	<0.001
**All Alarm Advisor**^b^	139682	139153	36545	38205	139682	133107	3.82 ± 0.03	3.64 ± 0.03	3.76–3.88	3.59–3.70	4.7%	<0.001
**MP Heart Rate**	36475	34576	36545	38205	36475	33074	1.00 ± 0.02	0.91 ± 0.01	0.97–1.03	0.88–0.93	9.3%	<0.001
**MP Respiratory Rate**	53404	49233	36545	38205	53404	47094	1.46 ± 0.02	1.29 ± 0.02	1.42–1.50	1.25–1.33	11.8%	<0.001
**MP SpO**_**2**_	40034	41722	36545	38205	40034	39909	1.10 ± 0.01	1.09 ± 0.01	1.07–1.12	1.07–1.12	0.3%	0.854
**MP Arterial**	9769	13622	1822	3021	9769	8216	5.36 ± 0.15	4.51 ± 0.09	5.07–5.67	4.33–4.69	15.9%	<0.001

SD, standard deviation. CI, confidence intervals.

^a^Data were weighted according to the number of overall monitoring hours per phase (Pre: 36545 hours, Post: 38205 hours). Arterial alarm data were weighted according to the number of arterial line monitoring hours per phase (Pre: 1822 hours, Post: 3021 hours).

^b^Consists of alarms addressed by Alarm Advisor: medium priority heart rate, respiratory rate, SpO_2_, and arterial blood pressure alarms.

Of the 5 alarm types that Alarm Advisor addresses (medium priority heart rate, respiratory rate, SpO_2_, arterial pressure, and PVCs per minute), we considered only the first 4 in our analyses. PVCs per minute were defaulted to generate only an inaudible visual alert before the start of the study. Our decision to exclude PVCs per minute from our analysis was justified by the finding that only 3 of these alerts were generated in the entire pre-intervention phase of the study.

**Fig 3:**
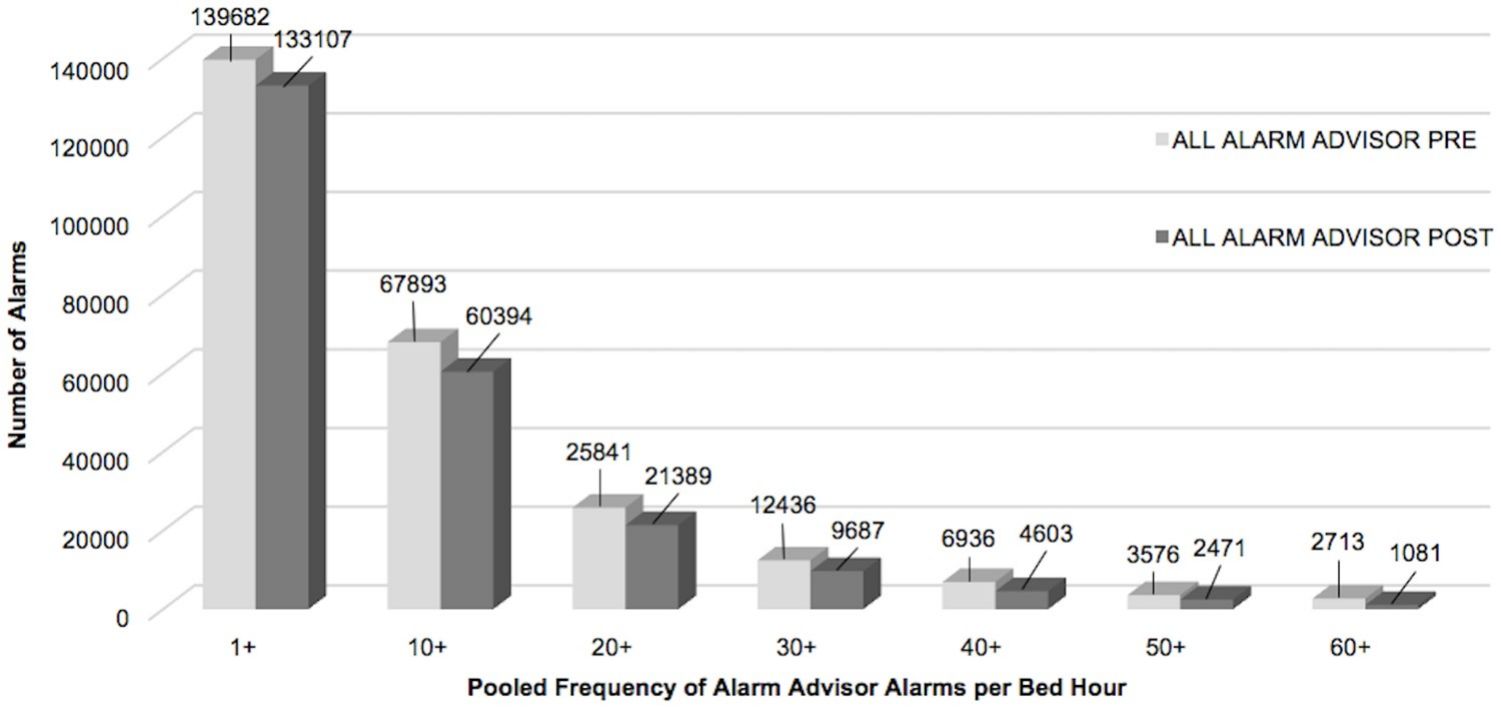
Alarms addressed by Alarm Advisor categorized by number of alarms/bed hour (1+–60+). Alarms addressed by Alarm Advisor: medium priority heart rate, respiratory rate, SpO_2_, and arterial pressure alarms.

**Fig 4:**
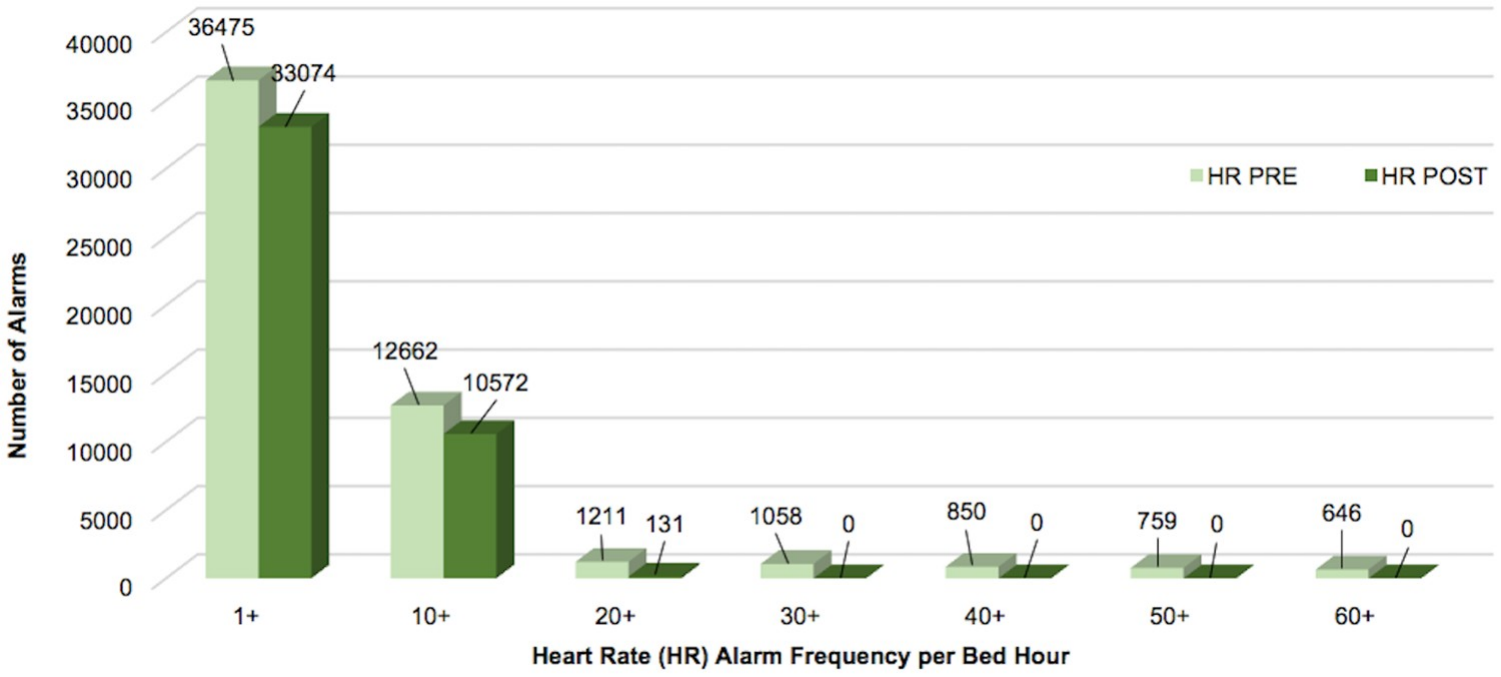
Medium priority heart rate alarms categorized by number of alarms/bed hour (1+–60+).

**Fig 5:**
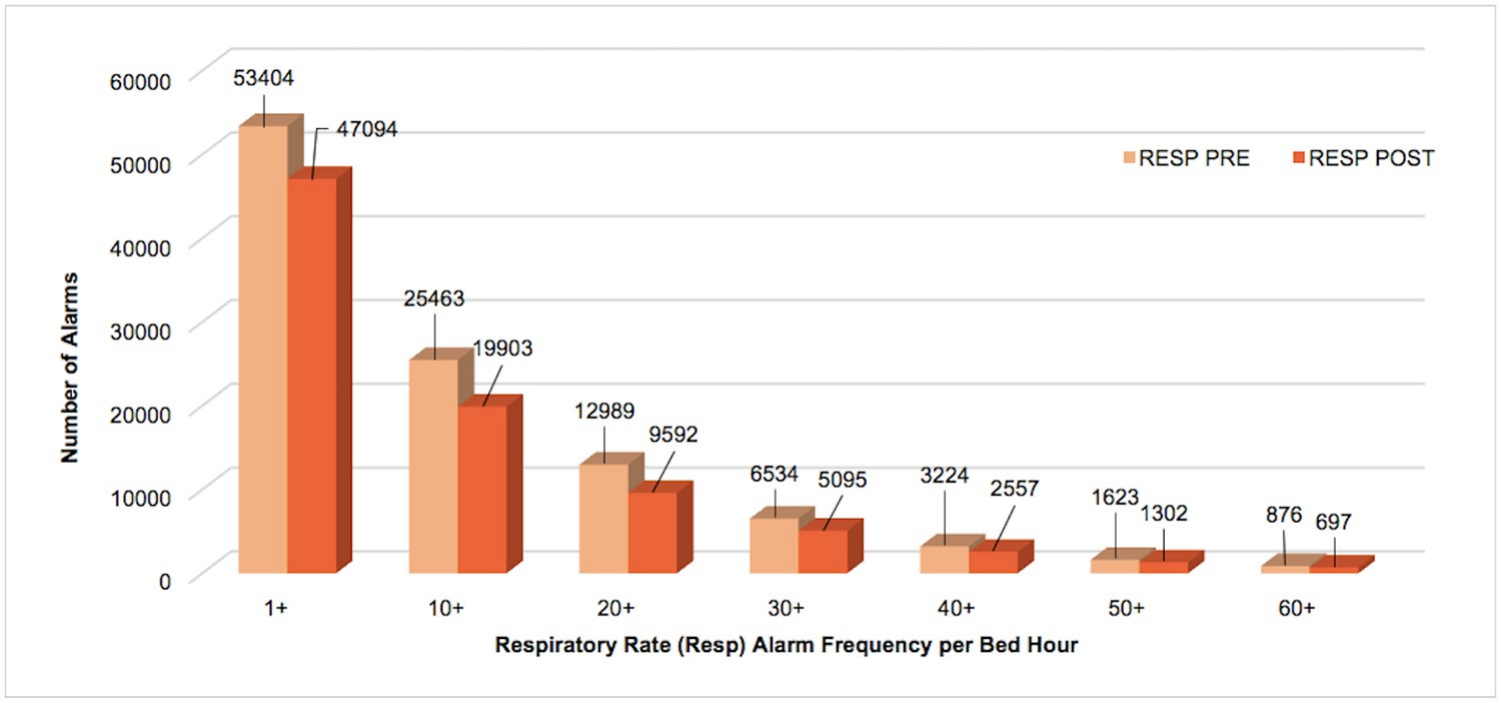
Medium priority respiratory rate alarms categorized by number of alarms/bed hour (1+–60+).

**Fig 6:**
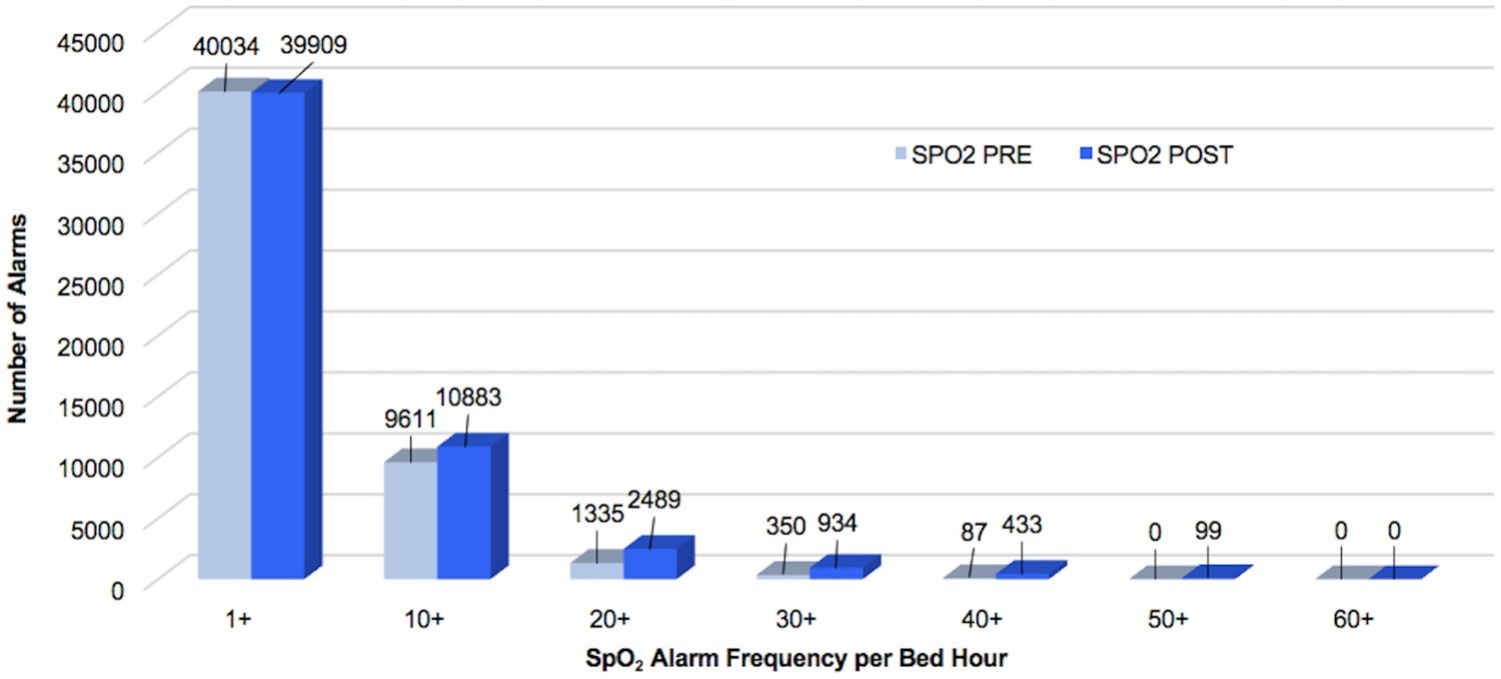
Medium priority SpO_2_ alarms categorized by number of alarms/bed hour (1+–60+).

**Fig 7:**
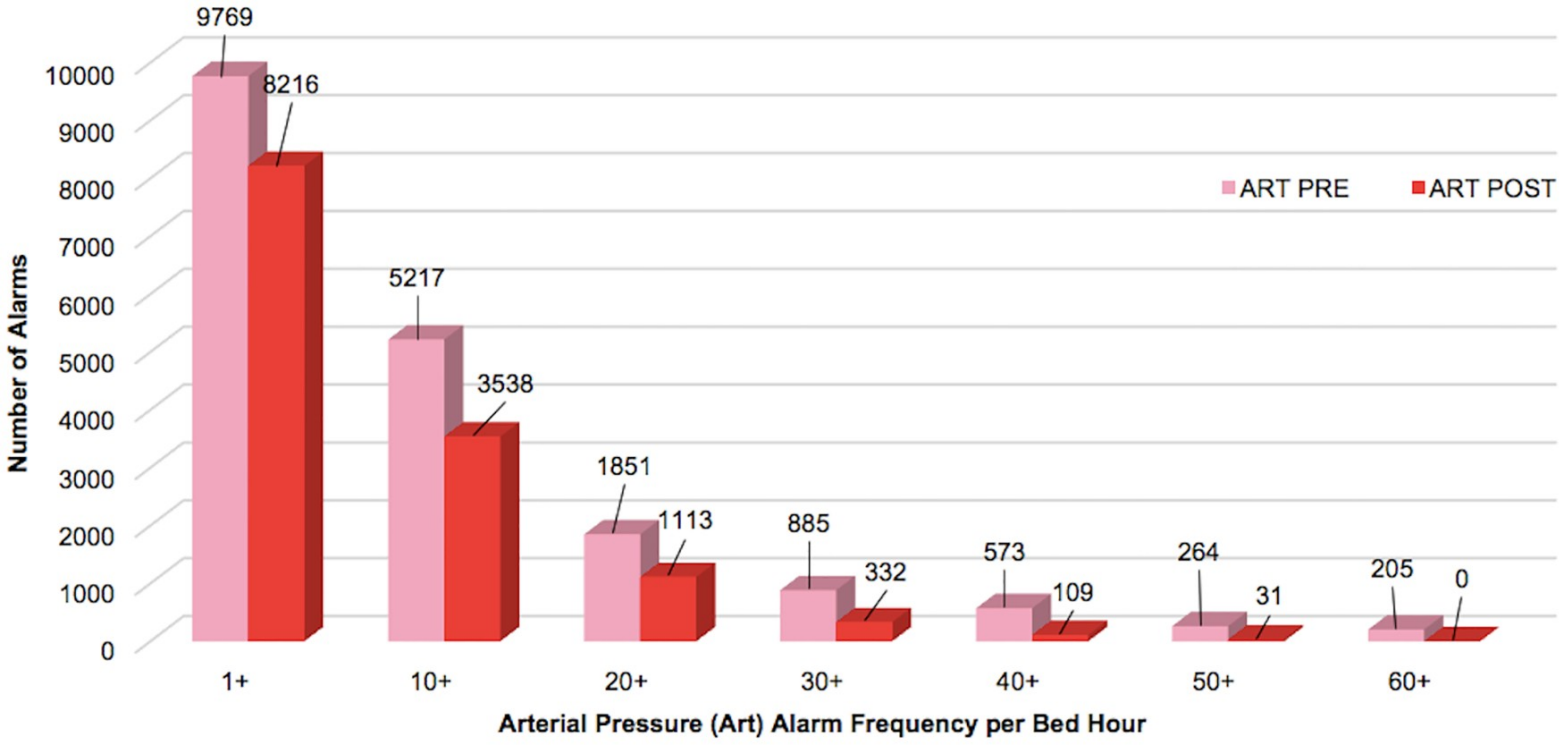
Medium priority arterial pressure alarms categorized by number of alarms/per bed hour (1+–60+).

Respiratory rate alarms were the most frequently occurring both pre- and post-implementation, followed by SpO_2_ alarms. When combined, the 4 alarm types addressed by Alarm Advisor had a statistically significant reduction in mean alarms per bed hour (4.7%, p<0.001). When we examined the weighted alarm data for the 4 alarm types individually, we found a significant reduction in heart rate alarms (9.3%, p<0.001), respiratory rate alarms (11.8%, p<0.001), and arterial pressure alarms (15.9%, p<0.001). SpO_2_ alarms were not significantly reduced (0.3%, p = 0.854). Figs 3–7 show alarm counts for the 4 alarm types together (Fig 3) and separately (Figs 4–7) pre- and post-implementation, at different frequency thresholds: 1+, 10+, 20+, 30+, 40+, 50+, and 60+ alarms per bed hour. No medium priority heart rate alarms occurred at a frequency of >30+ alarms per bed hour in the post-implementation phase (Fig 4).

The mean number of all medium priority alarms per bed hour significantly decreased pre- to post-implementation (p<0.001) (Table 3). However, this includes some types of alarms that do not generate Alarm Advisor alerts. The mean number of high priority alarms per bed hour was not significantly lower pre- to post-implementation; Alarm Advisor does not address these types of alarms.

Table 4 contains a comparison of the duration of alarms in the pre- and post-implementation phases. Again, we weighted the time in alarm based on the monitoring hours in each phase to facilitate comparison. When combined, the 4 alarm types addressed by Alarm Advisor had a statistically significant reduction in the mean time (minutes) in the alarm condition (6.0%, p<0.001). The duration in the alarm condition for heart rate and respiratory rate were significantly reduced in the post- implementation phase (7.8% and 13.3%, p<0.001). The duration of arterial pressure alarms was also significantly reduced (9.3%, p = 0.043), but no significant reduction in the duration of SpO_2_ alarms was found (0.7%, p = 0.788). The aggregate duration of all medium and high priority alarms was also not significantly reduced.

**Table 4:** Comparison of alarm duration pre- and post-intervention.

	Time in Alarm (minutes)		Monitoring Hours		Weighted Time in Alarm^a^ (minutes)		Bootstrap Means ± SD per Bed Hour (minutes)		Bootstrap 95% CI of Means (minutes)		Percent Reduction of Time in Alarm	Bootstrap p-value
*Alarm Types / Phase*	*Pre*	*Post*	*Pre*	*Post*	*Pre*	*Post*	*Pre*	*Post*	*Pre*	*Post*		
**All Medium and High Priority**	300181	313435	36545	38205	300181	299816	8.21 ± 0.08	8.20 ± 0.08	8.06–8.37	8.05–8.36	0.1%	0.926
**High Priority**	5008	5273	36545	38205	5008	5044	0.14 ± 0.00	0.14 ± 0.00	0.13–0.14	0.13–0.14	no reduction (-0.7%)	0.758
**All Medium Priority (MP)**	295174	308162	36545	38205	295174	294772	8.08 ± 0.08	8.07 ± 0.08	7.93–8.23	7.92–8.22	0.1%	0.920
**All Alarm Advisor**^b^	128033	125775	36545	38205	128033	120310	3.50 ± 0.04	3.29 ± 0.04	3.42–3.59	3.21–3.37	6.0%	<0.001
**MP Heart Rate**	103881	100138	36545	38205	103881	95787	2.84 ± 0.04	2.62 ± 0.04	2.76–2.92	2.55–2.70	7.8%	<0.001
**MP Respiratory**	8271	7493	36545	38205	8271	7168	0.23 ± 0.01	0.20 ± 0.00	0.22–0.24	0.19–0.21	13.3%	<0.001
**MP SpO**_**2**_	12337	12811	36545	38205	12337	12254	0.34 ± 0.01	0.34 ± 0.01	0.33–0.35	0.32–0.35	0.7%	0.788
**MP Arterial**	3545	5333	1822	3021	3545	3217	1.95 ± 0.07	1.77 ± 0.06	1.81–2.08	1.66–1.88	9.3%	0.043

SD, standard deviation. CI, confidence intervals.

^a^Data were weighted according to the number of the overall monitoring hours per phase (Pre: 36545 hours, Post: 38205 hours). Arterial alarm data were weighted according to the number of arterial line monitoring hours per phase (Pre: 1822 hours, Post: 3021 hours).

^b^Consists of alarms addressed by Alarm Advisor: medium priority heart rate, respiratory rate, SpO_2_, and arterial blood pressure alarms.

### Nurses’ experience of alarms

A total of 110 surveys were completed by 82 nurses. Twenty-eight nurses took the survey twice (pre- and post-implementation), and 54 nurses took the survey only once (pre- or post-implementation). Sixty-six nurses completed the survey in the pre-implementation phase (50.8% response rate) and 44 in the post-implementation phase (33.8% response rate). In the post-implementation phase nurses tended to have more years of medical ICU experience than nurses pre-implementation, but the difference was not statistically significant (pre-implementation: median 3.3, IQR 1.0–8.0, mean 6.2 years; post-implementation: median 5.0, IQR 2.0–15.0, mean 9.2 years; p = 0.054).

Survey results related to nurses’ experience of alarms are displayed in Table 5. We report on the survey questions directly related to Alarm Advisor; some additional questions on the survey focused on alarm management topics that were not addressed by Alarm Advisor and those are not reported here. We dichotomized Likert-style questions for the analyses (e.g., we compared those who agreed or strongly agreed with those who were neutral, disagreed, or strongly disagreed).

**Table 5:** Comparison of nurses’ experience of alarms pre- and post-intervention.

Survey Item	Pre (N = 66)	Post (N = 44)	P
Patient monitors on my unit are currently issuing too many alarms (Strongly agree or agree)	68.2%	54.5%	0.147^a^
I feel overwhelmed by too many alarms (Strongly agree or agree)	50.0%	47.7%	0.815^a^
The current alarm load on my unit disturbs my workflow (Strongly agree or agree)	66.7%	45.5%	0.027^a^
How much of your nursing time is consumed by responding to non-actionable alarms? (≥ 20%)	63.6%	41.9%	0.026^a^
How often do you adjust your patient’s alarm limits (Always or often)	69.7%	65.9%	0.676^a^
In the last 4 weeks, how often did you encounter a situation where a patient needed urgent attention, but no one responded to the alarm? (Often or sometimes)	43.9%	25.0%	0.043^a^
# of minutes spent handling unnecessary alarms for 1 patient per shift			0.995^b^
Median (IQR)	20.0 (10.0–30.0)	20.0 (10.0–30.0)	
Mean	28.2	25.2	

^a^Chi-square

^b^Wilcoxon-Mann-Whitney test

When comparing nurses’ responses (Table 5), we found a significant improvement from pre- to post-implementation of Alarm Advisor on 3 of the 7 items: agreement regarding the current alarm load on the unit disturbing workflow (from 66.7% to 45.5%, p = 0.027); spending at least 20% of time responding to non-actionable alarms (defined in the survey as an alarm that is accurate but not clinically meaningful) (from 63.6% to 41.9%, p = 0.026); and encountering a situation where a patient needed urgent attention and no one responded to the alarm (from 43.9% to 25.0%, p = 0.043). Evidence of improvement on other items did not reach statistical significance.

### Acceptance of Alarm Advisor

We added 6 questions to the post-implementation survey that addressed nurses’ use and acceptance of Alarm Advisor software (Table 6). Overall, participants were positive about the use of Alarm Advisor: more than half of the 43 respondents reported that they learned to use Alarm Advisor quickly, felt confident about using it, and believed that Alarm Advisor supported setting appropriate alarm limits. Only 1 respondent (2.3%) reported that the alarm-related workload went up with the use of Alarm Advisor. However, most (69.8%) rarely or never saw the Alarm Advisor alert window pop up for a patient and only 32.6% agreed or strongly agreed that Alarm Advisor helped to reduce non-actionable alarms on their unit.

**Table 6:** User acceptance of Alarm Advisor.

Survey Item	% (N = 43)
**How frequently did an Alarm Advisor window pop up for your patients?**	
Never	9.3%
Rarely	60.5%
About once per shift	18.6%
More than once per shift	11.6%
**How has your alarm-related workload changed with the use of Alarm Advisor?**	
Gone down	27.9%
Stayed the same	69.8%
Gone up	2.3%
**I figured out how to use Alarm Advisor quickly.**	
Strongly agree	14.0%
Agree	51.2%
Neither agree nor disagree	23.3%
Disagree	7.0%
Strongly disagree	4.7%
**I feel confident using Alarm Advisor.**	
Strongly agree	14.0%
Agree	51.2%
Neither agree nor disagree	23.3%
Disagree	9.3%
Strongly disagree	2.3%
**Alarm Advisor reduces non-actionable alarms in my unit.**	
Strongly agree	7.0%
Agree	25.6%
Neither agree nor agree	51.2%
Disagree	14.0%
Strongly disagree	2.3%
**Alarm Advisor supports me in setting appropriate alarm limits for my patient.**	
Strongly agree	4.7%
Agree	51.2%
Neither agree nor disagree	30.2%
Disagree	11.6%
Strongly disagree	2.3%

## Discussion

We examined a monitor software intervention to improve nurses’ awareness of alarm trends, in an effort to reduce alarm rates and improve nurses’ experience of alarms. We found a significant reduction in mean alarms per bed hour and alarm duration for medium priority heart rate, respiratory rate, and arterial pressure alarms. Although the unit on which we conducted the study had many alarm reduction strategies in place before this study, the presence of Alarm Advisor was associated with a further reduction in these alarms. Nurses did not perceive that Alarm Advisor increased their workload.

The only type of alarm affected by Alarm Advisor for which we did not find a significant reduction in count or duration was medium priority SpO_2_. We have considered several possible explanations. First, the post-implementation data collection occurred during peak influenza and respiratory virus season, which may have caused more SpO_2_ alarms than is typical. We did not find a statistically significant difference in frequency of primary respiratory diagnoses between patients in the pre- and post-implementation phases, but primary diagnosis provides only a limited picture of patient condition. Second, leaders on the unit had already implemented several specific interventions to address excessive and extraneous SpO_2_ alarms, including decreasing the lower alarm limit to 88% and extending the audible alarm delay from 10 to 15 seconds in the default configurations. Nurses may be uncomfortable further lowering the SpO_2_ limit. We found it surprising that despite the changes to SpO_2_ alarm configurations already made on the unit, there were still many SpO_2_ alarms overall. One potential explanation is poor signal quality from the oxygen saturation sensors, which would contribute to excessive false alarms. However, we were unable to assess whether alarms were valid or invalid (false) in this study, so we cannot confirm this speculation.

Although the reductions in mean alarm count and duration for medium priority heart rate, respiratory rate, and arterial pressure were statistically significant, we do not know if the reductions were clinically important. For example, the mean number of respiratory rate alarms per bed hour was 1.46 in the pre-implementation phase and 1.29 in the post-implementation phase. The threshold at which alarm fatigue develops is difficult, or impossible, to determine and the number of alarms that nurses consider to be disruptive to their workflow may fluctuate depending on contextual factors. For example, a nurse may consider a small number of alarms to be extremely disruptive when a patient is on isolation precautions compared with a patient for whom the nurse does not need to don a gown to enter the room. In addition to high average alarm rates, periods of excessive alarms per hour may also be disruptive to nurses. Therefore, it may be important to note that after implementing Alarm Advisor, no heart rate alarms occurred at a frequency of 30+ alarms per bed hour.

We attempted to measure the clinical significance of the reduction in alarms using the survey. We found significant reductions in nurses’ perceptions of how much time they spent responding to alarms and the frequency of urgent situations in which an alarm was ignored. These responses indicate that the reductions in alarms may have been perceptible to nurses. However, nurses’ perception of time is subjective, and we were unable to validate their experience with objective data because we did not measure response times to alarms.

We were unable to measure directly nurses’ exposure to Alarm Advisor alerts, but in the survey, nurses indicated that they rarely or never saw Alarm Advisor alerts for their patients. This may be because Alarm Advisor settings were not stringent enough to trigger alerts, even for alarms that were clinically irrelevant, or because nurses were already tailoring alarms appropriately for patients. Despite the seemingly low exposure to the intervention, the decrease in alarm rates and durations that we found appears promising because the monitoring hours actually increased in the post-implementation phase. Most of the alarms directly addressed by Alarm Advisor demonstrated significant reductions in rate and duration, whereas high priority alarms, which are not directly addressed by Alarm Advisor, did not significantly change pre-to-post-implementation.

Given nurses’ low exposure to Alarm Advisor, it is prudent to consider alternative explanations for the reduction in alarms that we found. The post-implementation data collection occurred in the winter when the unit traditionally experiences an increase in severity of patients’ illnesses and increase in census. Increased severity of illness was reflected by the lower patient GCS scores and increase in arterial pressure monitoring hours in the post-implementation phase. When patients are sicker, it is possible that nurses are more attentive to their alarms and adjust them more frequently. In addition, sicker patients are often less active, and so may not generate as many false alarms as a result of motion artifact.

Although strong observational alarm research exists [7, 14, 19], few rigorous intervention studies to address alarm fatigue have been undertaken in clinical settings [3]. Other investigators, primarily implementing quality improvement bundles, have attempted to demonstrate reduction in alarms using a pre/post design [8, 9, 13, 20, 21]. Although they found reductions in alarm rates after the intervention, comparing the magnitude of our results to other studies is challenging because of variation in the way alarm rates were measured and how alarm reduction was calculated. To our knowledge, no other intervention study has used weighted alarm data with bootstrapping in the statistical analysis of alarm reduction. Additionally, other investigators have not always compared patient characteristics in the pre- and post-implementation phases or performed statistical analyses of data.

### Limitations

In addition to these strengths, this study has several limitations. First are limits to generalizability of the findings. We conducted the study in 1 unit in 1 academic medical center and had a relatively low response rate of nurses to the survey (approximately half in the pre-implementation phase and one-third in the post-implementation phase). Because we obtained only gender, age, GCS score, and primary diagnosis, our comparisons of the pre- and post-implementation patient groups are limited. In addition, GCS scores were available for only 79% of the patients pre-implementation and 84% post-implementation. GCS scores are also a limited approximation of severity of illness. The unit had also undertaken extensive alarm reduction strategies before implementation of the Alarm Advisor software.

We were unable to obtain objective data on how frequently Alarm Advisor alerts were triggered or how nurses interacted with Alarm Advisor alerts (e.g., whether they customized alarm settings from the Alarm Advisor alert window). We did not gather any data on if and when nurses customized alarm settings or on nurses’ response times to alarms. We also could not categorize alarms by relevance or accuracy, so we could not determine if non-actionable alarms specifically were reduced. Although difficult to measure [22], the goal of alarm interventions should be to increase the proportion of alarms that are both accurate and relevant while also reducing the number of alarms overall. Our survey was not a validated instrument to measure the phenomenon of alarm fatigue, so we could not quantify or make objective statements regarding reduction in alarm fatigue. Because we used a pre/post design our study was subject to effects of history and maturation. Finally, Alarm Advisor software addressed only parameter limit alarms. We believe arrhythmia alarms, like atrial fibrillation and irregular heart rate alarms, are excessive and future customization support software should address these types of alarms.

## Conclusions

Implementing software to assist nurses with alarm customization was associated with a reduction in alarm rates and durations for 3 of the 4 types of alarms targeted by the software (medium priority heart rate, respiratory rate, and arterial pressure alarms). Nurses found Alarm Advisor easy to use and generally did not think that it increased their workload. The use of software to support nurses’ recognition of trends in patients’ alarms and facilitate changes to alarm settings may add a valuable component to alarm reduction initiatives. Further research is needed to examine the association of Alarm Advisor with alarm rates and nurse workload in more and different ICU settings.

## Supporting information

S1 Dataset Patient characteristics. (XLSX)

S2 Dataset Number of alarms. (XLSX)

S3 Dataset Duration of alarms. (XLSX)

S4 Dataset Nurse survey. (XLSX)

S1 Text Data dictionary. (DOCX)
