# Supplementary material for: Testing physiologic monitor alarm customization software to reduce alarm rates and improve nurses’ experience of alarms in a medical intensive care unit
Source: PLoS One. 2018 Oct 18;13(10):e0205901. doi: 10.1371/journal.pone.0205901 (PMC6193710; doi:10.1371/journal.pone.0205901)
Supplement: S1 Text — (DOCX) [file pone.0205901.s005.docx]

**S1 Data Dictionary**

1. **Patient Characteristics from Electronic Health Record**

| **Variable Name** | **Description** |
| --- | --- |
| Study ID | Randomly assigned unique patient identification number |
| Phase | Phase of study  Pre = pre-intervention, Post = post-intervention |
| Gender | Female, Male |
| Age | In years, age 90 or older aggregated into a single category (90) |
| Glasgow Coma Score (GCS) | GCS score – possible range 3-15 |

1. **Number of Alarms Per Hour**

| **Variable Name** | **Description** |
| --- | --- |
| ALL PRE | All medium & high priority alarms – pre-intervention |
| ALL POST | All medium & high priority alarms – post-intervention |
| HIGH PRE | All high priority alarms – pre-intervention |
| HIGH POST | All high priority alarms – post-intervention |
| MEDIUM PRE | All medium priority alarms – pre-intervention |
| MEDIUM POST | All medium priority alarms – post-intervention |
| SPO2 PRE | Medium priority SpO_2_ alarms – pre-intervention |
| SPO2 POST | Medium priority SpO_2_ alarms – post-intervention |
| HR PRE | Medium priority heart rate alarms – pre-intervention |
| HR POST | Medium priority heart rate alarms – post-intervention |
| RESP PRE | Medium priority respiratory alarms – pre-intervention |
| RESP POST | Medium priority respiratory alarms – post-intervention |
| ART PRE | Medium priority arterial pressure alarms – pre-intervention |
| ART POST | Medium priority arterial pressure alarms – post-intervention |
| ALL ALARM ADVISOR PRE | All alarms addressed by Alarm Advisor: medium priority heart rate, respiratory rate, SpO_2_, & arterial blood pressure alarms – pre-intervention |
| ALL ALARM ADVISOR POST | All alarms addressed by Alarm Advisor: medium priority heart rate, respiratory rate, SpO2, & arterial blood pressure alarms – post-intervention |

1. **Duration of Alarms Hourly in Seconds**

| **Variable Name** | **Description** |
| --- | --- |
| ALL PRE | All medium & high priority alarms – pre-intervention |
| ALL POST | All medium & high priority alarms – post-intervention |
| HIGH PRE | All high priority alarms – pre-intervention |
| HIGH POST | All high priority alarms – post-intervention |
| MEDIUM PRE | All medium priority alarms – pre-intervention |
| MEDIUM POST | All medium priority alarms – post-intervention |
| SPO2 PRE | Medium priority SpO_2_ alarms – pre-intervention |
| SPO2 POST | Medium priority SpO_2_ alarms – post-intervention |
| HR PRE | Medium priority heart rate alarms – pre-intervention |
| HR POST | Medium priority heart rate alarms – post-intervention |
| RESP PRE | Medium priority respiratory alarms – pre-intervention |
| RESP POST | Medium priority respiratory alarms – post-intervention |
| ART PRE | Medium priority arterial pressure alarms – pre-intervention |
| ART POST | Medium priority arterial pressure alarms – post-intervention |
| ALL ALARM ADVISOR PRE | All alarms addressed by Alarm Advisor: medium priority heart rate, respiratory rate, SpO_2_, & arterial blood pressure alarms – pre-intervention |
| ALL ALARM ADVISOR POST | All alarms addressed by Alarm Advisor: medium priority heart rate, respiratory rate, SpO2, & arterial blood pressure alarms – post-intervention |

1. **Nurse Survey**

| **Variable Name** | **Description** |
| --- | --- |
| Phase | Phase of study  1 = pre-intervention, 2 = post-intervention |
| StudyID | Randomly assigned unique nurse identification number |
| YrsinMICU | Years of experience in MICU (free-text) |
| TooManyAlarms | Patient monitors in my unit are issuing too many alarms.  1 = Strongly agree, 2 = Agree, 3 = Neither agree nor disagree, 4 = Disagree, 5 = Strongly disagree |
| TooManyAlarms2 | Patient monitors in my unit are issuing too many alarms. *(Dichotomized)*  1 = Strongly agree, Agree, 2 = Neither agree nor disagree, Disagree, Strongly disagree |
| Overwhelmed | I feel overwhelmed by too many alarms.  1 = Strongly agree, 2 = Agree, 3 = Neither agree nor disagree, 4 = Disagree, 5 = Strongly disagree |
| Overwhelmed2 | I feel overwhelmed by too many alarms.  *(Dichotomized)*  1 = Strongly agree, Agree, 2 = Neither agree nor disagree, Disagree, Strongly disagree |
| DisturbWork | The current alarm load on my unit disturbs my workflow.  1 = Strongly agree, 2 = Agree, 3 = Neither agree nor disagree, 4 = Disagree, 5 = Strongly disagree |
| DisturbWork2 | The current alarm load on my unit disturbs my workflow. *(Dichotomized)*  1 = Strongly agree, Agree, 2 = Neither agree nor disagree, Disagree, Strongly disagree |
| TimeRespNonactionable | How much of your nursing time is consumed by responding to non-actionable alarms?  1 = 0%, 2 = 10%, 3 = 20%, 4 = 30%, 5 = >30% |
| TimeRespNonactionable2 | How much of your nursing time is consumed by responding to non-actionable alarms? *(Dichotomized)*  1 = 0%, 10%, 2 = 20%, 30%, >30% |
| TimeUnnecAlarms | The average number of minutes I currently spend to handle unnecessary alarms for one patient per shift is:  (free text) |
| FreqAdjustAlarmLimits | How often do you adjust your patient’s alarm limits?  1 = Always, 2 = Often, 3 = Sometimes, 4 = Rarely, 5 = Never |
| FreqAdjustAlarmLimits2 | How often do you adjust your patient’s alarm limits? *(Dichotomized)*  1 = Always, Often, 2 = Sometimes, Rarely, Never |
| NotRespondtoAlarm | In the last 4 weeks, how often did you encounter a situation where a patient needed urgent attention but no one responded to the alarm?  1 = Often, 2 = Sometimes, 3 = Rarely, 4 = Never |
| NotRespondtoAlarm2 | In the last 4 weeks, how often did you encounter a situation where a patient needed urgent attention but no one responded to the alarm?  1 = Often, Sometimes, 2 = Rarely, Never |
| **Post-intervention Phase Only** | |
| FreqAAPopUp | How frequently have you seen an Alarm Advisor window pop up for your patients?  1 = Never, 2 = Rarely, 3 = About once per shift, 4 = More than once per shift |
| WorkloadChangeAA | Responding to non-actionable alarms may affect your workload. How has your alarm-related workload changed with the use of Alarm Advisor software?  1 = Gone down, 2 = Stayed the same, 3 = Gone up |
| UseAAQuickly | I figured out how to use Alarm Advisor quickly.  1 = Strongly agree, 2 = Agree, 3 = Neither agree nor disagree, 4 = Disagree, 5 = Strongly disagree |
| ConfidentUseAA | I feel confident using Alarm Advisor.  1 = Strongly agree, 2 = Agree, 3 = Neither agree nor disagree, 4 = Disagree, 5 = Strongly disagree |
| AAReduceNonactionableAlarms | Alarm Advisor reduces non-actionable alarms in my unit.  1 = Strongly agree, 2 = Agree, 3 = Neither agree nor disagree, 4 = Disagree, 5 = Strongly disagree |
| AASupportSettingLimits | Alarm Advisor supports me in setting appropriate alarm limits for my patient.  1 = Strongly agree, 2 = Agree, 3 = Neither agree nor disagree, 4 = Disagree, 5 = Strongly disagree |
